# Predictive and Prognostic Role of Pre-Therapy and Interim 68Ga-DOTATOC PET/CT Parameters in Metastatic Advanced Neuroendocrine Tumor Patients Treated with PRRT

**DOI:** 10.3390/cancers14030592

**Published:** 2022-01-25

**Authors:** Rexhep Durmo, Angelina Filice, Federica Fioroni, Veronica Cervati, Domenico Finocchiaro, Chiara Coruzzi, Giulia Besutti, Silvia Fanello, Andrea Frasoldati, Annibale Versari

**Affiliations:** 1 Nuclear Medicine Unit, Azienda USL-IRCCS of Reggio Emilia, 42123 Reggio Emilia, Italy; Angelina.Filice@ausl.re.it (A.F.); Chiara.Coruzzi@ausl.re.it (C.C.); Annibale.Versari@ausl.re.it (A.V.); 2 PhD Program in Clinical and Experimental Medicine (CEM), University of Modena and Reggio Emilia, 41125 Modena, Italy; 3 Medical Physics Unit, Azienda USL-IRCCS of Reggio Emilia, 42123 Reggio Emilia, Italy; Federica.Fioroni@ausl.re.it (F.F.); Domenico.Finocchiaro@ausl.re.it (D.F.); 4 Nuclear Medicine Unit, Azienda Ospedaliero-Universitaria di Parma, 43126 Parma, Italy; vcervati@ao.pr.it; 5 Radiology Unit, Azienda USL-IRCCS of Reggio Emilia, 42123 Reggio Emilia, Italy; Giulia.Besutti@ausl.re.it; 6 Medical Oncology Unit, Azienda USL-IRCCS of Reggio Emilia, 42123 Reggio Emilia, Italy; Silvia.Fanello@ausl.re.it; 7 Department of Endocrinology and Metabolism, Azienda USL-IRCCS of Reggio Emilia, 42123 Reggio Emilia, Italy; Andrea.Frasoldati@ausl.re.it

**Keywords:** neuroendocrine tumor, GEP-NET, PET/CT, PRRT, DOTATOC

## Abstract

**Simple Summary:**

Although a significant improvement has been achieved in the management of metastatic neuroendocrine tumor (NET), disease progression is observed in 20–30% of patients treated with peptide receptor radionuclide therapy (PRRT). Therefore, the early identification of patients who are at high risk of treatment failure is important to avoid futile therapy toxicities. The aim of this study was to identify biomarkers derived from baseline and interim 68Ga-DOTATOC PET/CT in patients undergoing PRRT. In 46 metastatic NET patients with available baseline and interim PET, only baseline total tumor volume (bTV) was able to discriminate responders to PRRT (partial response or stable disease) vs. non-responders. Patients with high bTV had also the worst overall survival. bTV, an imaging biomarker, integrated in the initial workup of NET patients could improve risk stratification and contribute to a tailored therapy approach.

**Abstract:**

Peptide receptor radionuclide therapy (PRRT) is an effective therapeutic option in patients with metastatic neuroendocrine tumor (NET). However, PRRT fails in about 15–30% of cases. Identification of biomarkers predicting the response to PRRT is essential for treatment tailoring. We aimed to evaluate the predictive and prognostic role of semiquantitative and volumetric parameters obtained from the 68Ga-DOTATOC PET/CT before therapy (bPET) and after two cycles of PRRT (iPET). A total of 46 patients were included in this retrospective analysis. The primary tumor was 78% gastroenteropancreatic (GEP), 13% broncho-pulmonary and 9% of unknown origin. 35 patients (76.1%) with stable disease or partial response after PRRT were classified as responders and 11 (23.9%) as non-responders. Logistic regression analysis identified that baseline total volume (bTV) was associated with therapy outcome (OR 1.17; 95%CI 1.02–1.32; *p* = 0.02). No significant association with PRRT response was observed for other variables. High bTV was confirmed as the only variable independently associated with OS (HR 12.76, 95%CI 1.53–107, *p* = 0.01). In conclusion, high bTV is a negative predictor for PRRT response and is associated with worse OS rates. Early iPET during PRRT apparently does not provide information useful to change the management of NET patients.

## 1. Introduction

Neuroendocrine tumors (NETs) are relatively rare neoplasms that originate from endocrine cells, mostly of the gastroenteropancreatic tract (GEP) and the pulmonary system. Due to their indolent natural course, NETs are identified as locally advanced or with distant metastasis in 40–50% of patients [1].

Peptide receptor radionuclide therapy (PRRT) is an effective systemic therapeutic option for patients with advanced, metastatic, or unresectable NETs with high somatostatin receptor (SSTR) expression. PRRT consists in the intravenous systemic administration of somatostatin analogs (SSAs) labeled with a βminus (β-) emitting radioisotope (90Y and 177Lu), that binds SSTRs overexpressed in tumors with high affinity and specificity. The compound is internalized by endocytosis and retained in lysosomes of cells allowing the delivery of cytotoxic radiation directly on target cells, therefore producing the breakdown of intracellular DNA chains and cell death [2]. Although a significant improvement has been reported in progression free survival (PFS) and overall survival (OS), disease progression is still observed in 20–30% of patients treated with PRRT [3]. Moreover, PRRT can be associated with hematologic, renal, and hepatic toxicities [4]. Therefore, the early identification of patients who are at high risk of treatment failure represents an unmet need.

Positron emission tomography/computed tomography with 68Ga-DOTA-labelled somatostatin analogues (68Ga-DOTA-peptide PET/CT) is the elective imaging technique for diagnosis and management of NETs [5]. In clinical practice PET/CT plays a pivotal role also for properly selecting patients as candidates for PRRT. 68Ga-DOTA-peptide PET imaging is used for both evaluation of somatostatin receptor expression and correct staging of disease [6].

In addition, PET derived parameters, such as the semiquantitative standardized uptake values (SUVs), have been widely studied as prognostic and predictive factors in NET patients treated with PRRT [7]. In several other malignancies, FDG-PET-derived metabolic tumor volume (MTV) and total lesion glycolysis (TLG), which is the product of SUVmean × MTV, have shown a major role in prognosis and treatment monitoring compared to SUVs parameters [8]. MTV and TLG provide a direct estimation of the whole-body tumor burden and have been validated as quantitative imaging biomarkers in several tumors [9]. Furthermore, early changes in tumor burdens are a promising index of response to treatment and could be the basis of a more individualized treatment approach [10,11].

Hence, the aim of this study is to evaluate the predictive and prognostic value of semiquantitative and volumetric parameters calculated from 68Ga-DOTATOC PET/CT performed before PRRT. Moreover, we assessed the potential role of the change (Δ) between baseline and interim PET parameters after two cycles of PRRT for early prediction of response.

## 2. Materials and Methods

### 2.1. Study Design

We conducted a retrospective subgroup analysis of patients enrolled in a prospective, monocentric, non-randomized phase II clinical trial (EudraCT:2013-002605-65). All patients signed a written informed consent form. For this study we included biopsy-proven, unresectable, metastatic GEP or bronchopulmonary or unknown primary site NET patients, who were treated with PRRT in our institution (Azienda USL-IRCCS of Reggio Emilia, Italy). All patients underwent screening with 68Ga-DOTATOC PET/CT to confirm the adequate expression of SSTR type 2 on tumor sites and an interim PET/CT scan 7 weeks after the second cycle of PRRT. The association between PET parameters and outcomes (PRRT response and OS) was evaluated.

### 2.2. Ga-DOTATOC PET/CT

A baseline 68Ga-DOTATOC PET/CT (bPET) 1–3 weeks before PRRT and after two cycles of PRRT (interimPET) was performed with a hybrid scanner (Discovery STE; GE Healthcare). Image acquisition started 60 ± 10 min after administration of 2 MBq/kg of 68Ga-DOTATOC. All DOTATOC-avid lesions were semiautomatically segmented by an experienced board-certified nuclear medicine physician (V.C.) using a commercial software (PET VCAR, GE Healthcare). Subsequently, all regions of physiological or non-disease related uptake were manually removed. From the remaining volume-of interests (VOIs), containing all 68Ga-DOTATOC avid tumor lesions, maximum standardized uptake value (SUVmax), mean standardized uptake value (SUVmean), the somatostatin receptor-derived tumor volume (TV) and total lesion SSR expression (Total Lesion Activity-TLA, defined as SUVmean × TV) were measured. Based on the experience of metabolic tumor volume in FDG PET/CT studies a threshold above 41% of SUVmax was used to calculate TV. The whole-body tumor volume (bTV) was calculated by summing TV measurements of all lesions in each patient. The whole-body TLA (bTLA) was calculated by summing TLA of all lesions. Also, the ratio of the lesion SUVmax to the SUVmax in the spleen (SUVratio T/S) was calculated. Differences (Δ) in SUVmax, SUVmean, bTV, bTLA and SUVratio T/S were evaluated by calculating the percentage in variation of each parameter using the following formula: Delta = (interimPET − bPET)/bPET × 100.

### 2.3. PRRT

PRRT was performed according to the joint International Atomic Energy Agency (IAEA; Vienna, Austria), European Association of Nuclear Medicine (EANM; Vienna, Austria), and Society of Nuclear Medicine and Molecular Imaging (SNMMI; Reston, VA, USA) practical guidance ([12]). Briefly, the inclusion criteria for treatment with PRRT were histologically confirmed, unresectable, metastatic GEP or bronchopulmonary or unknown primary site NET patients; high expression of somatostatin receptor on baseline 68Ga-DOTATOC PET/CT (defined as greater than or equal to that of normal liver); a glomerular filtration rate greater than 50 mL/min/1.73 m^2^; a white blood cell count greater than 2.5 × 10^9^/L; a platelet count greater than 90 × 10^9^/L; bilirubin levels less than 2.5 mg/dL and ECOG performance status ≤ 2. A fractionated treatment protocol, with an activity of 1850–2590 MBq 90Y-DOTATOC or 3700–5550 MBq 177Lu-DOTATOC per treatment cycle every eight weeks, aimed at four to six courses with an interval of eight weeks, was followed. The activity prescription was determined on the basis of the Biological Effective Dose (BED) delivered to kidneys and on the basis of the absorbed dose to bone marrow, considered as the main organs at risk. Dosimetry was scheduled during the first course of therapy after a therapeutic administration of ^177^Lu-DOTATOC. The cumulative dose limit to kidneys was set to 46 Gy of BED for patients with no risk factors, and at 28 Gy for patients with risk factors, while the absorbed dose limit to bone marrow was set to 2 Gy for both. Documented disease progression at any time led to the cessation of PRRT and the classification of the patient as having progressive disease.

### 2.4. Assessment of Treatment Response

Response assessment was performed three months after completion of PRRT courses. Combination of anatomical imaging (CT/MRI), functional (PET) and clinical data in a multidisciplinary tumor board setting was used to define response. Response Evaluation criteria in Solid Tumors (RECIST version 1.1) criteria was used in determining anatomical response to PRRT. PET response was assessed using the European Organization for Research and Treatment of Cancer (EORTC) criteria. For the study analysis, responders were defined as patients with a complete/partial response or stable disease, and non-responders had progressive disease.

### 2.5. Statistical Analysis

Quantitative variables were expressed as median with range or mean with SD. Categorical variables are presented with absolute and relative frequencies. Mann-Whitney U test was used for comparing continuous variables between responders and non-responders. Chi-square test was used to analyze differences in discrete variables between responders and non-responders. Binary logistic regression was performed to identify factors predictive of treatment. Receiver operating characteristics (ROC) analysis was performed to identify optimal cut-off values for PET/CT quantitative parameters. The area under the curves (AUC), sensitivity, specificity and accuracy were reported. Survival curves were estimated according to the Kaplan-Meier method. A log-rank test was used to compare survival curves. Cox proportional-hazards model was used for univariate and multivariate survival analysis and results were reported as hazard ratio (HR), 95% confidence interval (95%CI) and *p*-Value based on statistical Wald-test. OS was defined as the time (months) from the first PRRT cycle to death from any cause. Surviving patients were censored at the last date of follow-up. All statistical tests were two-sided and *p* < 0.05 was considered a statistically significant result. Statistical analysis was performed using IBM SPS Statistics 25 (IBM, New York, NY, USA) and MedCalc Statistical Software 14.8.1 (Ostend, Belgium) for Windows.

## 3. Results

### 3.1. Patients

A total of 46 patients with available bPET and iPET were included in this retrospective analysis. The median age was 60 (range 25–85) and 21 patients (46%) were female. Primary tumor was for 78% GEP, 13% broncho-pulmonary and 9% of unknown origin. All patients had stage IV disease. Liver and lymph nodes were the most frequent sites of metastasis. Patients’ main characteristics are reported in Table 1.

### 3.2. PRRT and PET/CT

Forty-three patients (94%) received four to sixcycles of PRRT. Five patients (11%) were treated with 177Lu-DOTATOC radiopeptide, while the 41 remaining subjects underwent a combination of 90Y-DOTATOC and 177Lu-DOTATOC therapy.

The observed response rates were 21% (*n* = 10) partial response, 55.1% (*n* = 25) stable disease, and 23.9% (*n* = 11) progressive disease for the entire cohort. Considering GEP NET cohort response rates were 27% (*n* = 10) partial response, 51% (*n* = 18) stable disease, and 22% (*n* = 8) progressive disease. Complete response was not observed. For the analysis of this study, 35 patients (76.1%) with either stable disease or partial response after PRRT were classified as responders and 11 (23.9%) patients with progressive disease as non-responders.

**Figure 1:**
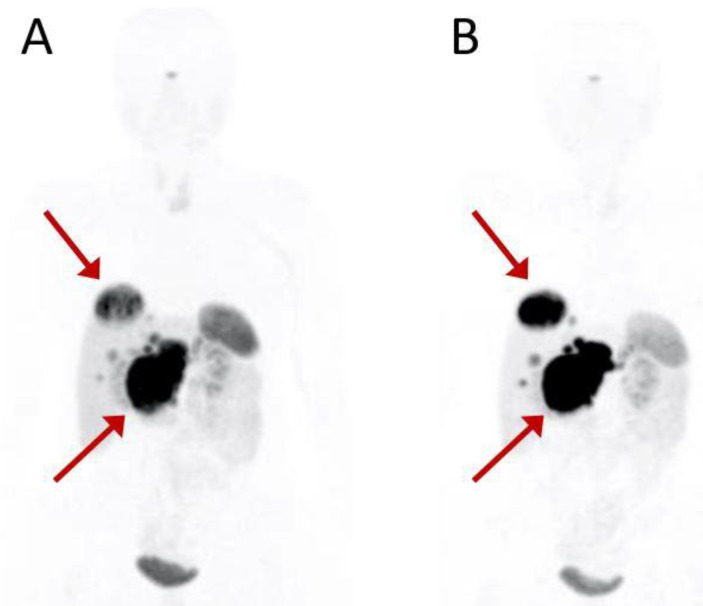
Baseline (**A**) and interim (**B**) PET scan of a 54-year-old patient with G1 pancreatic NET. Red arrows indicate the primary pancreatic tumor and the most representative liver metastasis. Interim 68Ga-DOTATOC PET after the second PRRT cycle shows disease progression. The patient was treated with a total of six PRRT courses (four cycles of 177Lu-DOTATOC + 2 cycles of 90Y-DOTATOC) for a total of 28,534 MBq activity and classified as partial response at the assessment following treatment.

The median values of SUVmax, SUVmean, SUVt/s, bTV and bTLA were 34 (IQR, 23.1–55.8), 9.9 (IQR, 7.6–16.4), 1.1 (IQR, 0.7–2.3), 143.8 mL (IQR, 32.9–354), and 1834 (IQR, 342–6309), respectively. The median percentage in variation between baseline and interimPET after two cycles of PRTT reported as ΔSUVmax, ΔSUVmean, ΔSUVratioT/S, ΔbTV, ΔbTLA were 1.1% (IQR, −21.2–22.1), 4.2% (IQR, −17.8–39.4), −1.1% (IQR, −41.5–24.5), 32.4% (IQR, −10.2–70.6), 25% (−12.5–80.9), respectively. Figure 1 shows a baseline and interim PET.

**Figure 2:**
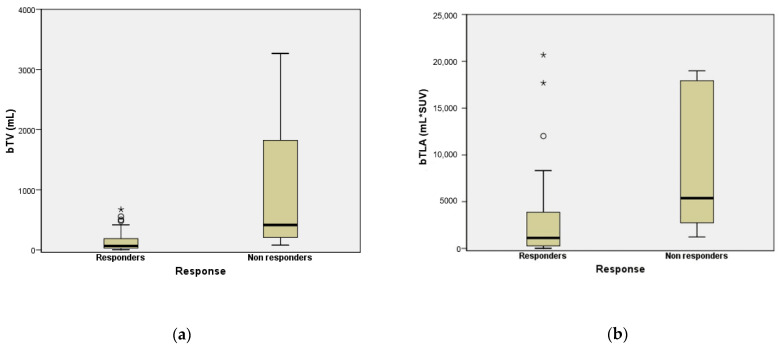
Box Plot comparing bTV (**a**) and bTLA (**b**) between PRRT responder and non-responder patients. “★’’ and “○’’ symbols represent outlier cases.

The bTV and bTLA values of patients that did not respond to PRRT were significantly higher than those of patients with response to PRRT therapy (496.2 IQR 218.3–2029.4 vs. 77.6 IQR 31–186.6, *p* < 0.001 and 6078.3 IQR 2813–18,959 vs. 1341 IQR 272.3–3865, *p* = 0.001, respectively: Figure 2).

No association between semiquantitative PET parameters and response were observed. Percentage variations (Δ) of PET semiquantitative and volumetric parameters were not significantly different between PRRT responders and non-responders. PET parameters and comparison between responders vs. non-responders are summarized in Table 2.

Logistic regression analysis of bPET derived parameters and ΔPET values identified that only bTV was associated with therapy outcome (OR 1.17; 95%CI 1.02–1.32; *p* = 0.02). No significant association with PRRT response was observed for other variables (Table 3).

### 3.3. Overall Survival Analysis

During the follow-up period (mean 31 months; ranged from eight to 86 months), seven (15.2%) patients died. Mean survival time was 69.4 (SD, 5.3) months, the median was non reached for the entire cohort.

ROC analysis showed a best cut-off point for bTV of >244.5 mL (sensitivity 85.7%; specificity 79.5%) and AUC of 0.77 (95% CI 0.62–0.88, *p* = 0.036) for identification of patients with worse OS. For bTLA best cut-off point was 2659 (sensitivity 85.7%; specificity 64.1%) and AUC of 0.71 (95% CI 0.56–0.84, *p* = 0.092). The complete ROC curves analysis of PET parameters is shown in Table S1.

**Figure 3:**
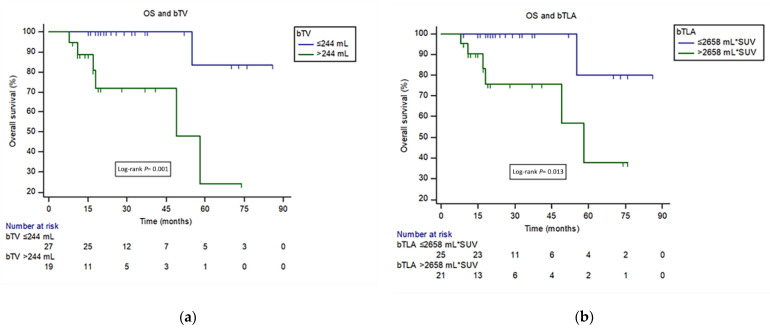
(**a**) Kaplan-Meier curves for overall survival (OS) among patients with high- and low-bTV defined on the basis of the ROC curve (244 mL). (**b**) Kaplan-Meier curves for OS among patients with high- and low-bTLA defined on the basis of the ROC curve (2658 mL*SUV).

In univariate analysis, higher bTV and bTLA were associated with lower survival probability (HR 13 95%CI 2.6–64.1, *p* = 0.001; HR 9.08 95%CI 1.09–75.76, *p* = 0.04) (Figure 3).

Moreover, a difference in SUVmax above 5.5% between iPET and bPET (ΔSUVmax) was associated with a favourable outcome (HR 0.15 95% CI0.03–0.67; *p* = 0.04). In Cox multivariate analysis, only a high bTV was confirmed as an independent prognostic factor (HR 12.76 95%CI 1.53–107, *p* = 0.01) (Table 4).

## 4. Discussion

After about 20 years of retrospective and phase I/II trials, PRRT was finally approved by regulatory authorities. Since its approval for the treatment of inoperable or metastatic GEP-NETs with progressive disease by the European Medicine Agency in 2017, PRRT has been widely used [13,14]. The phase III NETTER-1 trial has established that PRRT prolongs PFS compared to high dose octreotide LAR and improved quality of life. [3,15] In our study, 76.1% of NET patients had stable disease or partial response and 23.9% patients showed progressive disease after PRRT. These findings are in line with the response rates reported in a recent large meta-analysis where the disease control rate (complete or partial response and stable disease) was 83% in metastatic NET patients treated with PRRT [16]. Despite the increasing role of PRRT in metastatic NETs, what is clear both from our results and literature is that we need parameters to improve the selection of patient candidates to PRRT.

In this study, we evaluated the predictive and prognostic significance of semiquantitative and volumetric baseline PET imaging parameters in patients with advanced, metastatic NET undergoing PRRT. We also investigated the role of interim PET after two cycles of PRRT as an early predictor.

Several retrospective studies have assessed the role of semiquantitative parameters, mostly SUVmax, as prognostic markers for NETs. However, conflicting results have been published regarding the role of SUV on baseline 68Ga-DOTA-SSA PET in PRRT response prediction. Several authors demonstrated that a high SUVmax in baseline PET is associated with a favorable outcome and a wide range of different cut-off values are reported to separate responders to PRRT vs. non responders [17,18,19,20].

In our cohort we did not find any association between SUVs obtained in the baseline PET and PRRT response. This finding is in line with published results by Gabriel et al. and Soydal et al., who similarly found that SUVs showed no additional value for PRRT response prediction [21,22]. This controversial result may be because SUVs are measured in a single region of interest and thus they are not representative of the total tumor burden. Indeed, the lesion heterogeneity in NET patients, and the well-known limits of semiquantitative parameters, might further limit SUVs utility [23].

We found that patients who did not respond to PRRT had significantly higher bTV compared to responders. Moreover, bTV was the only PET parameter that confirmed a predictive role for PRRT response on logistic regression analysis (OR 1.17). Furthermore, bTV was an independent prognostic factor associated with worse OS rates in Cox regression analysis.

To our knowledge, only two other studies have previously evaluated the role of baseline volumetric PET parameters in patients treated with PRRT. Ohlendorf et al. found that PFS was shorter in patients with high bTV and high bTLA in 32 NET patients treated with PRRT [22]. Pauwels et al. reported that a bTV higher than 578 mL was associated with worse OS [23]. In our study population, the bTV cut-off that better identified patients with shorter OS was 244 mL. This difference in cut-offs could be explained by different segmentation methods. Indeed, Pauwels used a SUV threshold customized per patient through visual inspection to segment the tumor. In contrast, we used a semiautomatic method applying a threshold above 41% of the SUVmax for calculation of TV and TLA, based on the experience with FDG PET studies [24]. Further studies are warranted to define and harmonize 68Ga-Dota-peptide PET/CT tumor burden segmentation.

Our findings are in line with several previous studies that assessed the prognostic significance of PET volumetric parameters in settings other than PRRT [25,26,27,28,29,30]. Thus, bTV seems to be a powerful prognostic parameter in NET patients. However, bTV should be validated in further prospective studies including more homogeneous populations in terms of primary site, disease course and treatment setting.

In contrast, we did not find any relationship between changes in PET semiquantitative and volumetric parameters between baseline and interim PET after two cycles of PRRT. However, changes in SUVmax (ΔSUVmax) showed a significance in OS univariate analysis (*p* = 0.04) that was not confirmed in multivariate analysis. These results, in accordance with previous studies, show no utility of iPET in guiding the management of metastatic NET patients treated with PRRT [11,27].

There are several limitations of the present study that should be acknowledged. First are the retrospective nature and the small sample size. Second, we included patients with different NET sites. Furthermore, we did not evaluate FDG PET findings in our analysis. It is known that some patients with grade 2–3 NET can show higher affinity for FDG rather than 68Ga-DOTATOC PET [28]. This can result in an underestimation of the actual tumor burden by 68Ga-DOTATOC PET. A combination of these two tracers can be considered to address this issue. In addition, it should be noted that the generalizability of our findings with 68Ga-DOTATOC to the other often used 68Ga-DOTATATE PET ligand need to be confirmed. Indeed, while 68Ga-DOTATATE shows only affinity to SSTR2, 68Ga-DOTATOC also exhibits some affinity to SSTR5. However, despite differences in receptor affinities, a head-to-head comparison showed no clinically significant difference between the two tracers [29]. Finally, it is worth mentioning that other PET tracers like 64Cu-DOTATATE, providing better resolution and potentially a better lesion detection rate than 68Ga-DOTATOC, may improve tumor burden quantification [30]. However, the 64Cu-labeled tracers are not in use in the clinical routine and require further work.

## 5. Conclusions

In metastatic NET patients addressed to PRRT, higher total body tumor volume measured from baseline 68Ga-DOTATOC PET/CT scans is a negative predictor for therapy response and is associated with worse OS rates. Interim 68Ga-DOTATOC PET/CT after two cycles of PRRT did not allow the identification of patients with poorer prognosis that would justify a change in treatment strategy.

## Figures and Tables

**Table 1:** Main characteristics of the study population.

Patients	(*n* = 46)
Age, median (range) in years	60 (25–85)
Gender, *n* (%)	
Male	25 (54%)
Female	21 (46%)
Primary tumour, *n* (%)	
GEP	36 (78%)
Stomach	3 (8%)
Pancreas	8 (22%)
Intestine	25 (70%)
Broncho-Pulmonary	6 (13%)
Unkown	4 (9%)
GEP NETs WHO grade, *n* (%)	
G1 ((Ki-67 0–2%)	21 (46%)
G2 (Ki-67 3–20%)	19 (41%)
G3 (Ki-67 >20%)	2 (4%)
NA	4 (9%)
Metastasis, *n*	
Liver	21
Lymph nodes	23
Bone	15
Lung	7
Other	9
Cycles of PRRT, *n* (%)	
2	2 (4%)
3	1 (2%)
4	5 (11%)
5	11 (24%)
6	27 (59%)
Type of PRRT	
Only 177Lu	5 (11%)
177Lu + 90Y	41(89%)

Abbreviations: GEP: gastroenteropancreatic; PRRT: peptide receptor radionuclide therapy; NET: neuroendocrine tumor.

**Table 2:** Characteristic of calculated PET parameters. Data of all patients (*n* = 46) and a comparison between responders (*n* = 35) vs. non-responders (*n* = 11) groups are reported. Values are expressed in mean (SD) and median (IQR). Mann-Whitney U test was used to compare variables. The *p*-Values shown in boldface correspond to *p* < 0.05.

PET Parameters	All (*n* = 46)	Responders (*n* = 35)	Non-Responders (*n* = 11)	*p* Value
SUVmax				0.58
Mean (SD)	40 (24.9)	41.5 (27.1)	35.5 (35.5)	
Median (IQR)	34 (23.1–55.8)	34.5 (23.7–56.2)	33.5 (21.8–53.9)	
SUVmean				0.57
Mean (SD)	11.6 (5.9)	10.3 (4.1)	11.9 (6.3)	
Median (IQR)	9.9 (7.6–16.4)	9.9 (7.2–17.3)	10(7.8–13.5)	
SUVratio T/S				0.93
Mean (SD)	1.8 (0.9)	1.4 (0.7)	1.9 (2.2)	
Median (IQR)	1.1 (0.7–2.3)	1.2 (0.6–2.3)	1 (0.8–2.2)	
bTV				**<0.001**
Mean (SD)	371 (665.5)	143.7 (177.6)	1073.5 (1061,4)	
Median (IQR)	143.8 (32.9–354)	77.6 (31–186.8)	496.2 (218.3–2029.4)	
bTLA				**0.001**
Mean (SD)	5339.5 (8171.4)	3108.13 (4971.1)	12,236.4 (11,959)	
Median (IQR)	1834 (342–6309)	1341 (272.3–3865)	6078.3 (2813–18,959)	
ΔSUVmax				0.89
Mean (SD)	23.9 (117.7)	25.2 (129,6)	18.1 (62)	
Median (IQR)	1.1 (−21.2–22.1)	−2.5 (−21.2–22.2)	3.5 (−26.6–22.1)	
ΔSUVmean				0.84
Mean (SD)	25.5 (79.2)	45.6 (128)	19 (55,9)	
Median (IQR)	4.2 (−17.8–39.4)	6.2 (−16.5–25.4)	−10.2 (−27.4–47.6)	
ΔSUVratioT/S				0.42
Mean (SD)	1.3 (2)	1.9 (0.8)	1.4 (0.9)	
Median (IQR)	−1.1 (−41.5–24.5)	−11.5 (−40.5–20.9)	1.9 (−46.8–32.3)	
ΔTV				0.51
Mean (SD)	61 (170)	27.1 (55,6)	71.5 (756,7)	
Median (IQR)	32.4 (−10.2–70.6)	32.4 (−5.5–69.2)	32.4 (−23.9–79.9)	
ΔTLA				0.92
Mean (SD)	143 (668,4)	45.7 (79,1)	171.9 (756.7)	
Median (IQR)	25 (−12.5–80.9)	24.3 (−0.4–80)	36 (−22.5–111)	

Abbreviations: SUV: standardized uptake value; T/S: tumor/spleen; TV: total volume; TLA: total lesion activity.

**Table 3:** Logistic Regression Analysis.

PET Parameters	Univariate Analysis	
	OR (95%CI)	*p* Value
SUVmax	1.06 (0.99–1.13)	0.09
SUVmean	0.8 (0.55–1.15)	0.23
SUVratio T/S	0.84 (0.52–1.35)	0.48
bTV	1.17 (1.02–1.32)	**0.02**
bTLA	0.99 (0.93–1.01)	0.08
ΔSUVmax	0.97 (0.93–1.01)	0.25
ΔSUVmean	1.007 (0.98–1.01)	0.259
ΔSUVratioT/S	0.99 (0.98–1.01)	0.839
ΔTV	0.99 (0.98–1.01)	0.922
ΔTLA	0.99 (0.98–1.01)	0.70

Abbreviations: SUV: standardized uptake value; T/S: tumor/spleen; TV: total volume; TLA: total lesion activity. The *p*-Values shown in boldface correspond to *p* < 0.05.

**Table 4:** Univariate and multivariate Cox regression analysis for OS.

PET Parameter	Univariate Analysis		Multivariate Analysis	
	HR (95%CI)	*p* Value	HR (95%CI)	*p* Value
SUVmax (<22.02)	0.99 (0.12–8.20)	0.99	-	-
SUVmean (≤5.45)	0.31 (0.06–1.51)	0.26	-	-
SUVratioT/S (≤1.31)	0.84 (0.08–8.23)	0.87	-	-
bTV (>244.48)	13 (2.6–64.1)	**0.001**	12.76(1.53–107)	**0.01**
bTLA (>2658.62)	9.08 (1.09–75.76)	**0.04**	7.15 (0.96–68.1)	0.98
ΔSUVmax (>5.5598)	0.15 (0.03–0.67)	**0.04**	0.17 (0.02–1.46)	0.1
ΔSUVmean (>24.4984)	0.65 (0.05–7.87)	0.68	-	-
ΔSUVratioT/S (>−0.75)	0.14 (0.017–1.28)	0.08	-	-
ΔTV (≤−15.876)	0.23(0.02–1.86)	0.354	-	-
ΔTLA (>80.2)	0.45 (0.082–2.48)	0.2737	-	-

Abbreviations: SUV: standardized uptake value; T/S: tumor/spleen; TV: total volume; TLA: total lesion activity. The *p*-Values shown in boldface correspond to *p* < 0.05.

## Data Availability

The data presented in this study are available on request from the corresponding author. The data are not publicly available due to privacy-related issues.

## Supplementary Materials

The following are available online at https://www.mdpi.com/article/10.3390/cancers14030592/s1, Table S1: ROC curve analysis for OS.
