# Supplementary material for: Predictive and Prognostic Role of Pre-Therapy and Interim 68Ga-DOTATOC PET/CT Parameters in Metastatic Advanced Neuroendocrine Tumor Patients Treated with PRRT
Source: Cancers (Basel). 2022 Jan 25;14(3):592. doi: 10.3390/cancers14030592 (PMC8833820; doi:10.3390/cancers14030592)
Supplement: Supplementary file 1 [file cancers-14-00592-s001.zip › cancers-1539335-supplementary.pdf]

Supplementary materials: Predictive and Prognostic Role of Pre-Therapy and Interim 68Ga-DOTATOC PET/CT Parameters in Metastatic Advanced Neuroendocrine Tumor Patients Treated with PRRT

Rexhep Durmo, Angelina Filice, Federica Fioroni, Veronica Cervati, Domenico Finocchiaro, Chiara Coruzzi, Giulia Besutti, Silvia Fanello, Andrea Frasoldati and Annibale Versari

Table S1. ROC curve analysis for OS.

Variable	AUC	95%CI	*p*	Youden Index	Sens	Spec
**SUVmax**	0.64	0.49–0.78	0.24	<22.02	57.1	82
**SUVmean**	0.656	0.501 to 0.789	0.1971	≤5.45	42.86	92.31
**SUVratio T/S**	0.564	0.408 to 0.711	0.6552	≤1.3161	83.33	46.15
**bTV**	0.769	0.622 to 0.880	0.0368	>244.48	85.71	79.49
**bTLA**	0.714	0.562 to 0.838	0.0928	>2658.62	85.71	64.10
**ΔSUVmax**	0.740	0.590 to 0.858	0.0266	>5.5598	85.71	74.36
**ΔSUVmean**	0.656	0.501 to 0.789	0.2530	>24.4984	57.14	76.92
**ΔSUVratioT/S**	0.590	0.433 to 0.734	0.5121	>–0.7501	83.33	58.97
**ΔTV**	0.557	0.403 to 0.703	0.6770	≤-15.876	42.86	82.05
**ΔTL**	0.560	0.406 to 0.706	0.7248	>80.2123	57.14	82.05
